# Human T-cell Lymphotropic Virus Type I Associated with Amyotrophic Lateral Sclerosis Syndrome: Immunopathological Aspects and Treatment Options

**DOI:** 10.7759/cureus.7531

**Published:** 2020-04-03

**Authors:** Fernando Gonzalez Trujillo, Karen Parra Cortes, Yolima Álvarez Pareja, Jose Onate

**Affiliations:** 1 Neurology, Clínica de Occidente, Jamundi, COL; 2 Medicine, Clínica de Occidente, Jamundi, COL; 3 Internal Medicine, Clínica de Occidente, Jamundi, COL

**Keywords:** htlv-1, viral transmission, amyotrophic lateral sclerosis, tsp/ham, treatments

## Abstract

Human T-cell lymphotropic virus type I (HTLV-I) is a retrovirus related to infectious myelopathies, neoplasms, lymphomas, leukemias, and amyotrophic lateral sclerosis (ALS). It is acquired through sexual transmission, transfusion of blood products, and breastfeeding. The increased expression of human endogenous retrovirus K (HERV-K) in the brain tissue of patients with ALS has been demonstrated, a finding that supports the relationship between the virus and this disease. Therapeutic options include supportive measures and symptomatic treatment with anti-inflammatory medications including steroids, cyclosporines, pentoxifylline, danazol, interferons, and vitamin C. New management proposals are being implemented with valproic acid that acts to facilitate the recognition of the virus by the immune system and with zidovudine antivirals focused on reducing viral load. The purpose of this paper is to describe a clinical case that exhibits clinical signs and evidence of motor neuron compromise as described in electrophysiology studies along with positive laboratory tests for the HTLV-I virus.

## Introduction

The human T-cell lymphotropic virus type I (HTLV-I) is a retrovirus related to clinical cases of infectious myelopathy, which are of slow progression generating a spastic paraparesis with bladder dysfunction in patients [1]. It is acquired through sexual transmission, transfusion of blood products, and breastfeeding [1]. Up to 61 different syndromes have been described in connection with this retrovirus, including neoplasms, lymphomas, leukemias, and amyotrophic lateral sclerosis (ALS) [1,2]. The literature describes three probable scenarios to explain the etiology of tropical spastic paraparesis (TSP) and associated HTLV-I myelopathy: 1) from toxins, 2) toxic agents, and 3) even from the direct involvement of the HTLV-I virus, in cases where the astrocytes and the blood-brain barrier were affected and excitotoxicity was generated by glutamate in the cortical spinal pathways [1-3]. Currently, two hypotheses are proposed when the symptoms of myelopathy and ALS affect the patient: 1) the cause is co-incidental and the inflammatory process in the hypoandrogen-metabolic (HAM) syndrome would modify the symptoms of the ALS, and 2) the direct inflammation by the virus [2]. The virus infects CD4+ T cells by generating reactive processes such as cytokine secretion, presence of membrane-specific receptors for the virus, autocrine stimulation, lymphocyte activation, and production of autoantibodies [4,5]. Autopsy reports of patients who had mixed signs of the syndromes have shown infiltration with inflammatory cells, reduction and degeneration of the neural cells in the dorsal column, brainstem, cerebellum, and absence of the “Bunina” bodies that are used to identify ALS in pathology samples [2].

The purpose of the paper is to describe a case report that we find interesting due to the following reasons: the patient hails from a geographical area that this pathology is endemic to; the patient exhibits clinical signs and evidence of motor neuron compromise as revealed by electrophysiology studies along with positive laboratory tests for the HTLV-I virus.

## Case presentation

A 49-year-old female patient, from Buenaventura (Valle del Cauca, Colombia), with no relevant medical history, presented with progressive clinical symptoms of pain and weakness for nine months. The symptoms had originated in the lower limbs, and there was subsequent involvement of the upper limbs until progressively leading to quadriparesis. She had experienced dysarthria, swallowing disorder, and progressive loss of head and body support until being confined to bed with dyspnea and complete loss of autonomy and mobility.

**Figure 1:**
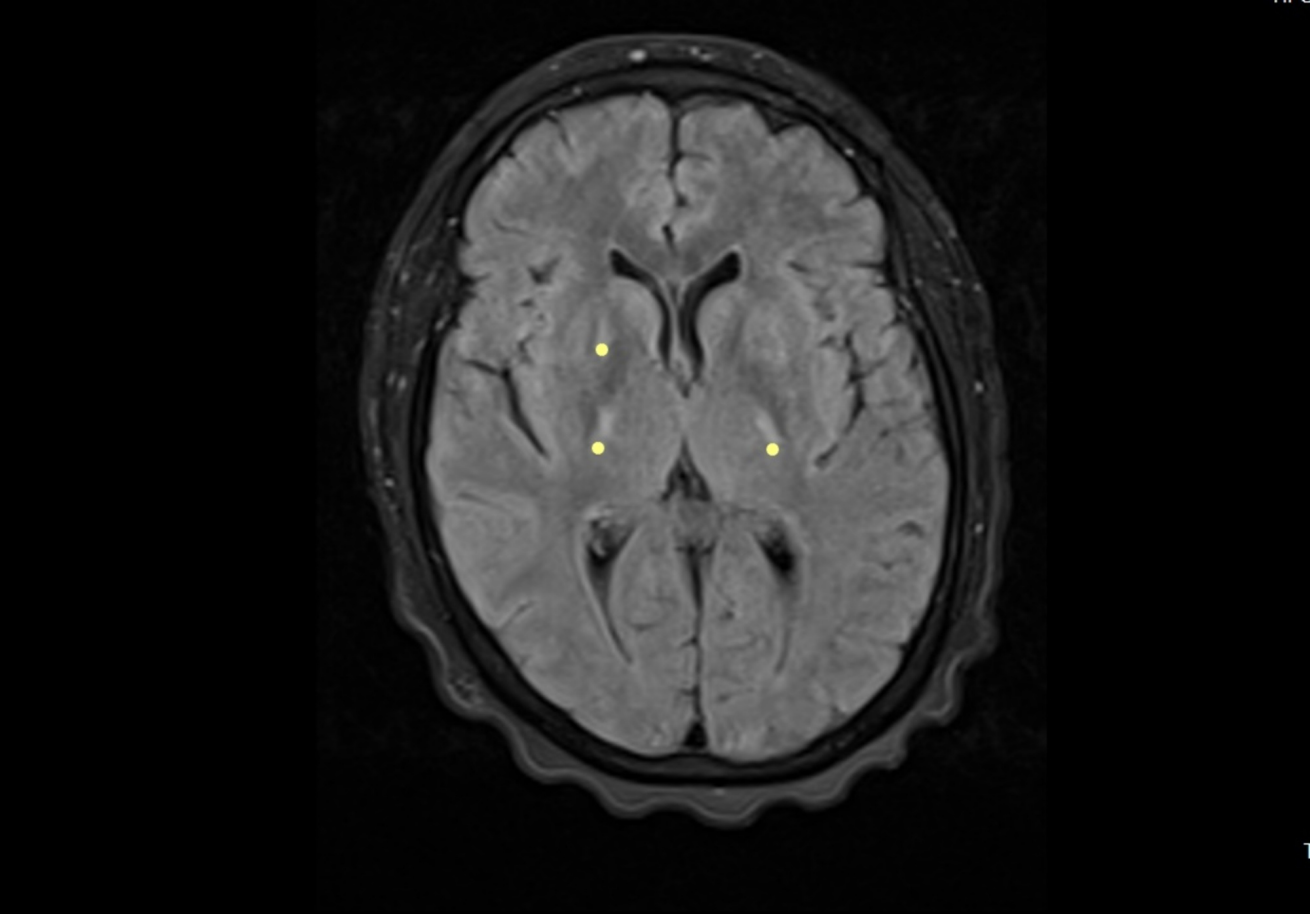
Brain MRI - view 1 FLAIR axial and T2-weighted coronal image. The image shows hyperintensity in the path of the corticospinal tract (yellow dots) MRI: magnetic resonance imaging; FLAIR: fluid attenuation inversion recovery

**Figure 2:**
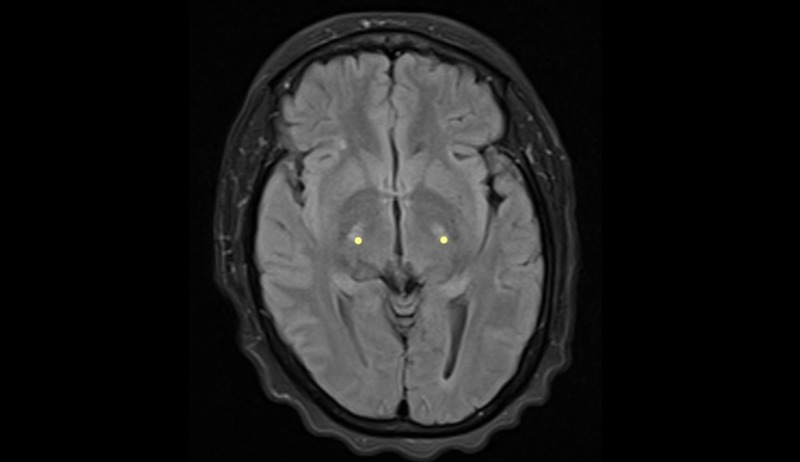
Brain MRI - view 2 FLAIR axial and T2-weighted coronal image. The image shows hyperintensity in the path of the corticospinal tract (yellow dots) MRI: magnetic resonance imaging; FLAIR: fluid attenuation inversion recovery

**Figure 3:**
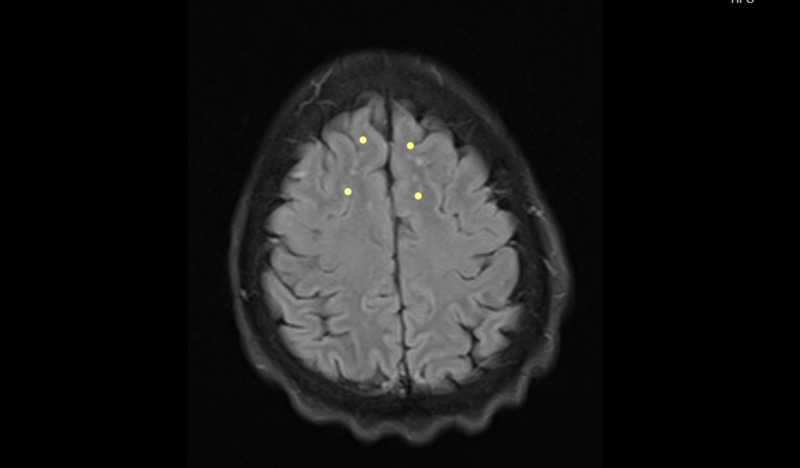
Brain MRI - view 3 FLAIR axial and T2-weighted image. The image shows hyperintensity in the path of the corticospinal tract (yellow dots) MRI: magnetic resonance imaging; FLAIR: fluid attenuation inversion recovery

**Figure 4:**
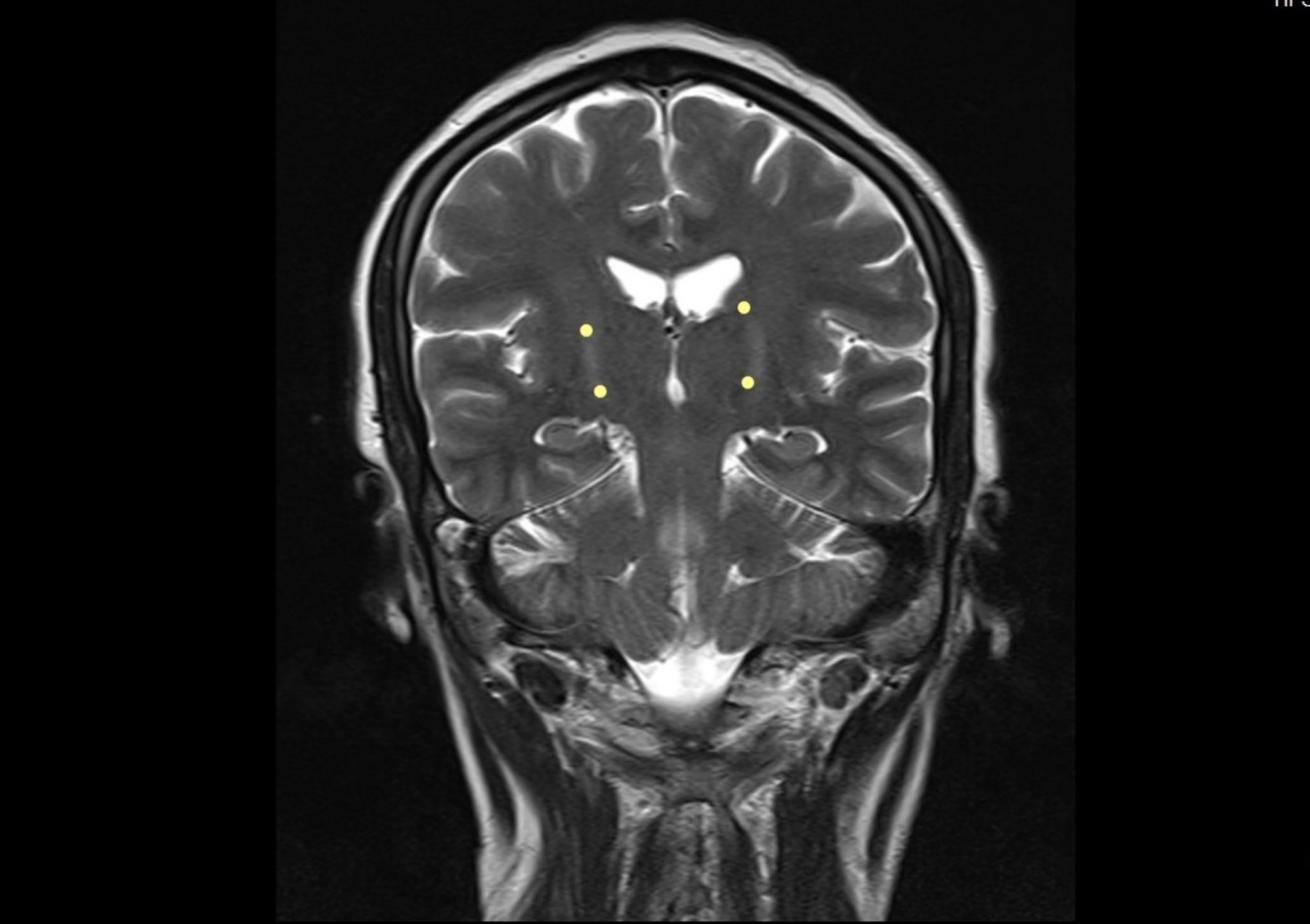
Brain MRI - view 4 FLAIR axial and T2-weighted coronal image. The image shows hyperintensity in the path of the corticospinal tract (yellow dots) MRI: magnetic resonance imaging; FLAIR: fluid attenuation inversion recovery

**Figure 5:**
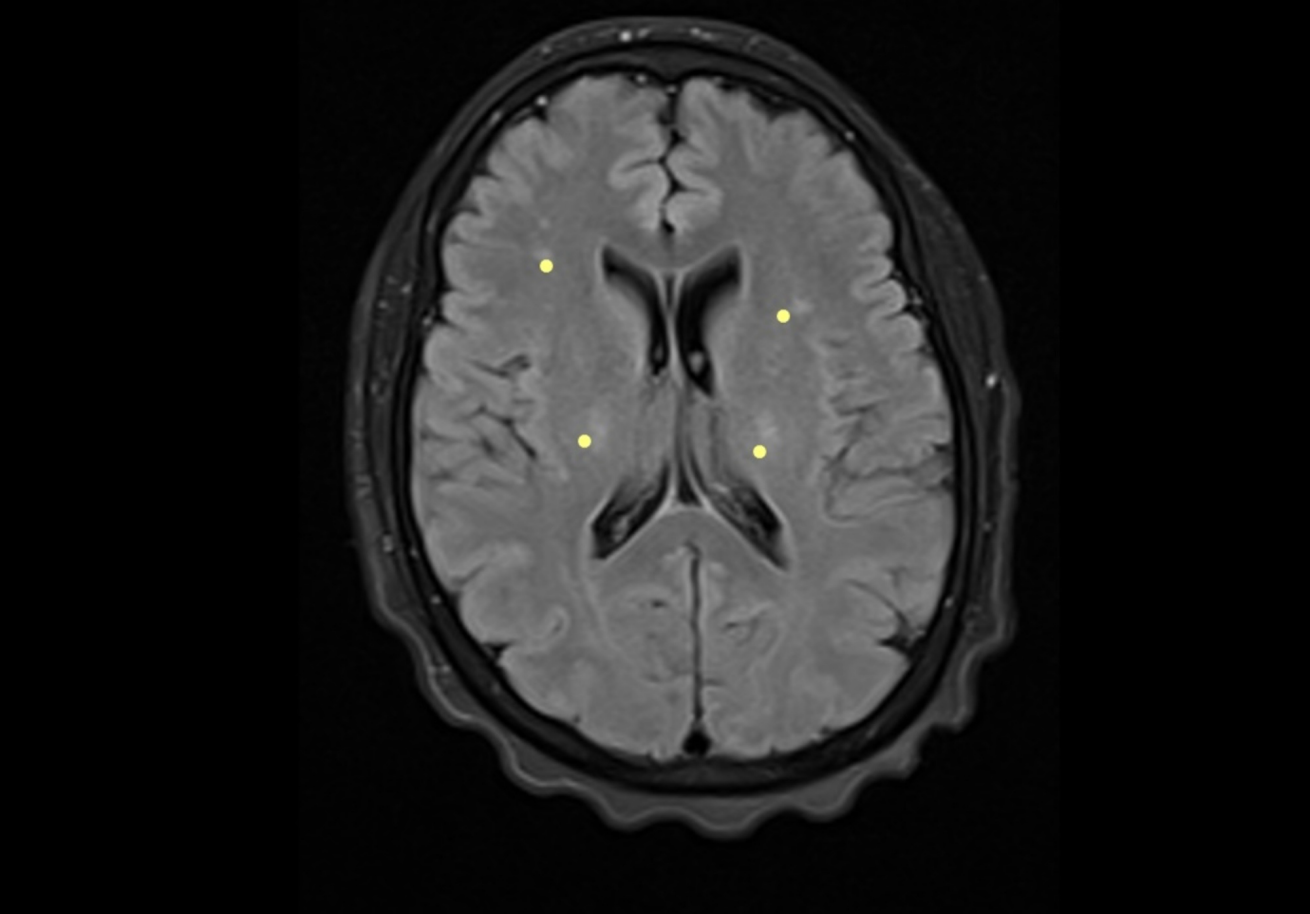
Brain MRI - view 5 FLAIR axial and T2-weighted coronal image. The image shows hyperintensity in the path of the corticospinal tract (yellow dots) MRI: magnetic resonance imaging; FLAIR: fluid attenuation inversion recovery

The physical examination revealed regular clinical conditions, sores, signs of respiratory difficulty, lack of head support, with swallowing reflex present but with dysfunction of her own secretions, preserved lingual protrusion, and presence of lingual fasciculations. There were quadriparesis with exalted reflexes, positive bilateral Hoffman´s sign, and muscular atrophy with spasticity. She did not have a sensitive commitment. She had a dysfunctional gait, and she remained in bed-chair. Extensive studies were performed, which returned a normal metabolic profile (glycemia, thyroid function, folic acid, and cyanocobalamin), negative infectious profile (serology: VDRL, HIV; hepatitis, tuberculosis), and a digestive tract study with normal endoscopy. The relevant reports were as follows: elevated tests of liver function and normal hepatobiliary ultrasound; chest radiography and total abdominal ultrasound ruled out the commitment of the reticuloendothelial system. The serum HTVL-I test was positive, and the cerebrospinal fluid (CSF) titers were negative. Brain MRI studies showed the presence of hyper-intensities in the corticospinal tract and changes in the cervicothoracic spine tracts by spondyloarthritis and local degenerative changes without compromise in the spinal cord (Figures 1-5).

**Figure 6:**
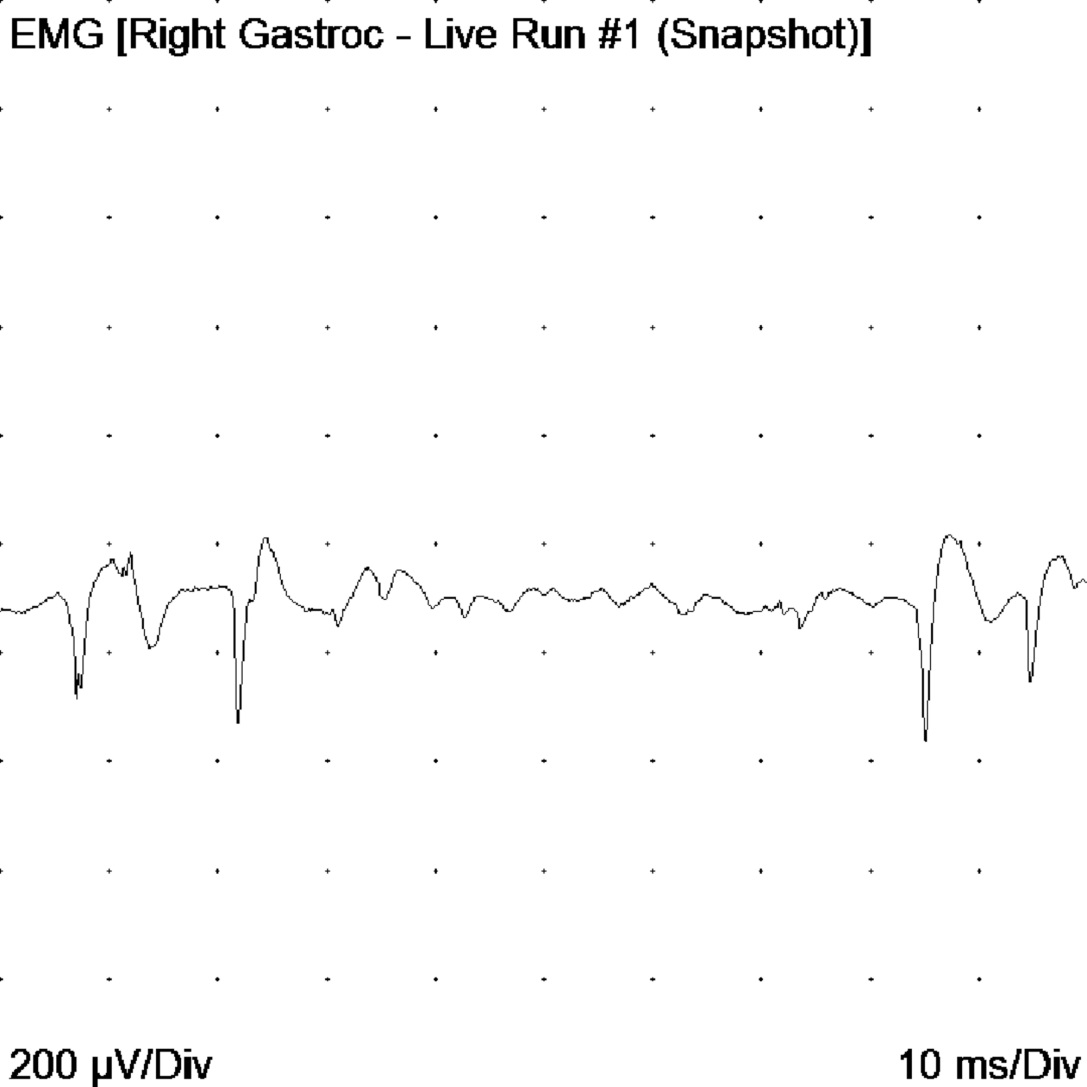
Acute positive waves in the right gastrocnemius muscle EMG: electromyogram

**Figure 7:**
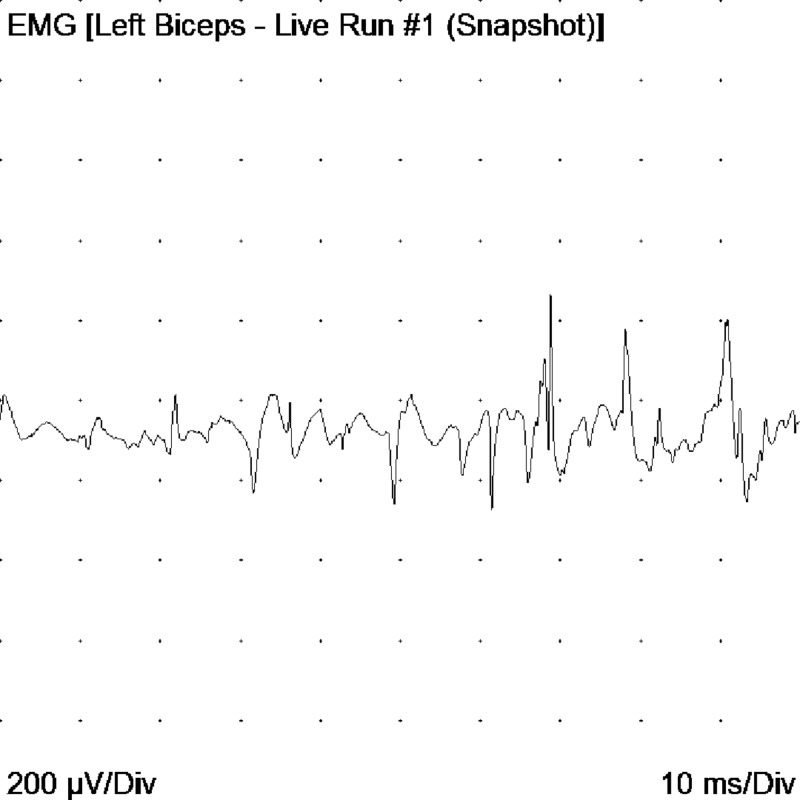
Acute positive waves and fibrillation potentials in left biceps brachial muscle EMG: electromyogram

**Figure 8:**
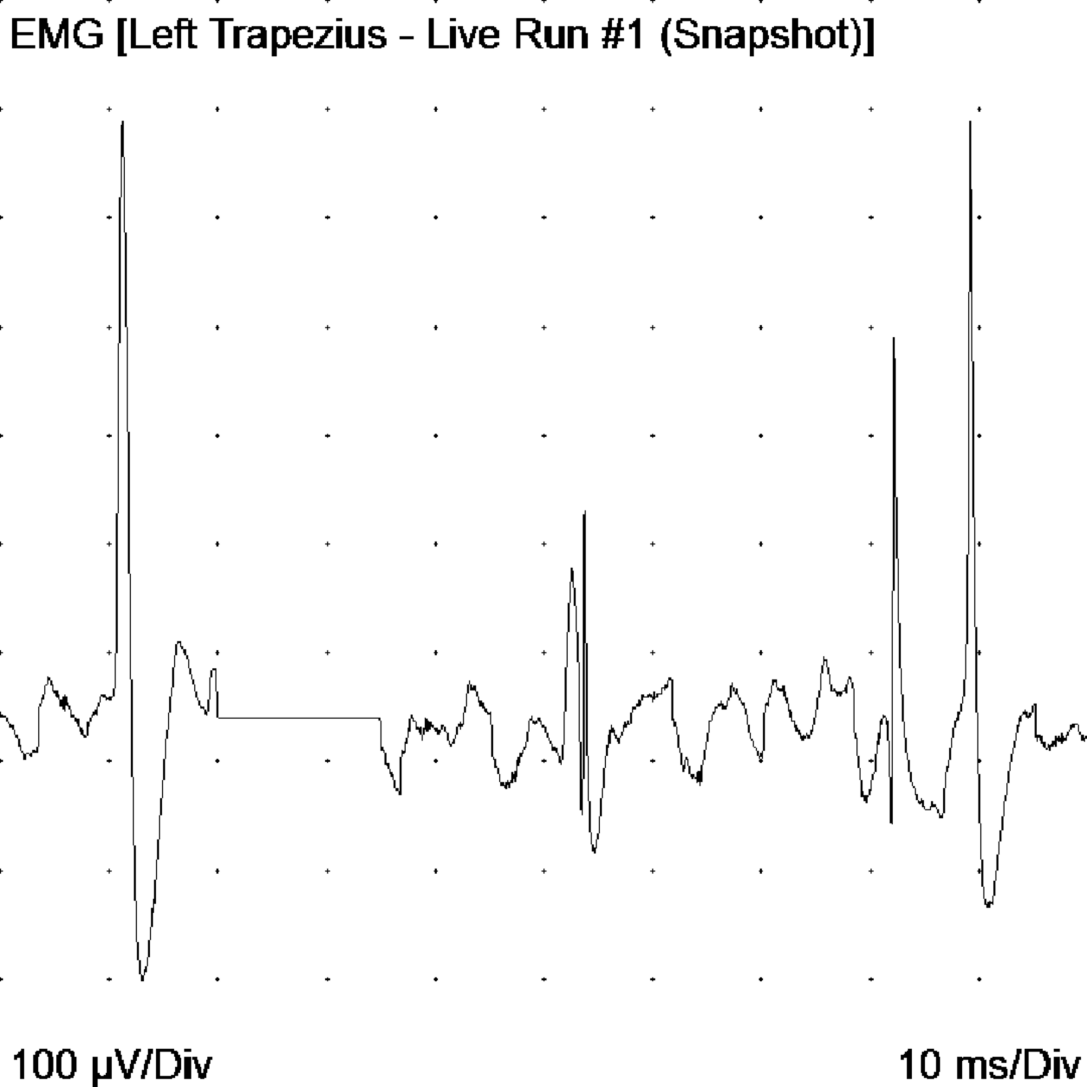
Decrease in recruitment and interference pattern in the left trapezius EMG: electromyogram

**Figure 9:**
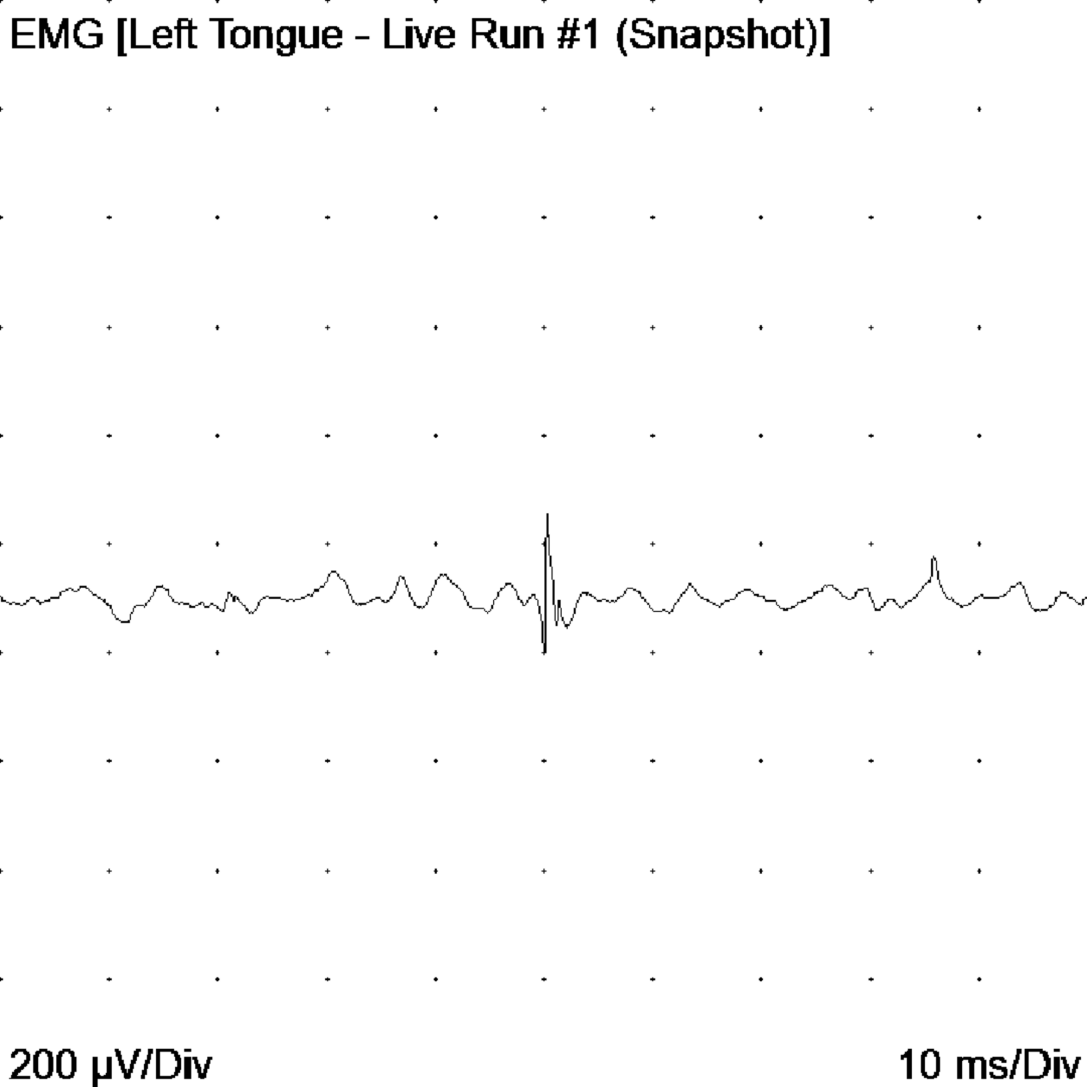
Fibrillation potentials found on the left side of the tongue EMG: electromyogram

The study by electrophysiology led to the following findings: motor neuro conductions of the median, ulnar, peroneal, and bilateral tibial nerves had decreased amplitudes and some distal latencies and slightly decreased conduction velocities. The electromyography with a monopolar needle showed abundant signs of denervation in muscles of upper and lower limbs, tongue, trapezius, and cervical para-spinal. Fasciculations were documented in the tongue; the findings were interpreted as indicative of motor neuron disease (Figures 6-9).

The patient was evaluated by neurology and Infectiology; the origin of the patient from an area where the virus is endemic to, the positivity of the serum markers, the findings suggestive of probable motor neuronal disease, and the referred diagnostic studies were reviewed. It was concluded that the patient was in the asymptomatic carrier phase for the HTLV-I virus; the causal relationship of the clinical syndrome with the virus was not ruled out, and there was evidence from imaging studies and electrophysiology of the probable motor neuron involvement. It was proposed to start treatment with riluzole, boluses of methylprednisolone 1 g intravenous per day for three days, followed by prednisone 25 mg orally, and gabapentin-type pain modulators in case of partial improvement of the symptoms. Support was provided with rehabilitation and integral care of the patient and discharge was given.

## Discussion

Our patient presented a rapidly progressive compromise in nine months, which severely limited functionality. Her condition was clinically aggressive from the beginning, which contrasted with the forms of ALS as referenced in the literature, which begins by engaging the lower limbs and evolve slowly for up to three years [3]. The clinical signs found led to a probable diagnosis of motor neuron disease, which was supported by paraclinical tests such as the resonance, where the presence of hyperintensities was observed in the corticospinal tracts, a finding usually described in the resonance images in patients with this pathology. The study by electrophysiology revealed the denervation signs present in different body muscle segments including tongue fasciculations, and it was considered that she met the diagnostic criteria for ALS. It was complemented with a large paraclinical study that was normal, except for the serological study for HTLV-I retrovirus, which was positive in serum and negative in CSF. It was concluded by the treating groups that the patient met the diagnostic criteria for motor neuron disease and that the probable causal relationship of the presence of the virus with the syndrome in the patient could not be ruled out. We provided therapeutic relief by prescribing steroids and riluzole, and moderate improvement was observed during hospitalization; however, the observation time (follow-up) was too short to establish results. We believe that evolution over time should be followed up to better assess the responses to therapeutic intervention.

Patients infected by the virus can remain in the state of being asymptomatic carriers for a long time. There are no specific biomarkers to establish the symptomatic state, and the measurement of high viral loads is considered a risk factor for progression [4]. Studies of antibodies against HTVL-I virus are performed in serum and CSF; this marker is important to differentiate between patients with high viral load and ALS-like symptoms directly affected by the virus and patients who are asymptomatic carriers with low viral load [1,2,4-6].

Retroviruses exhibit marked reactivity in cases with ALS, and it is supposed to explain the existence of human endogenous retrovirus (HERV), which are classified into different families, the most studied being HERV-K and HERV-W. Douville et al. demonstrated the increased expression of HERV-K in the brain tissue of patients with ALS, which varied according to the area of the brain involved: greater reactivity was found in the pre-frontal cortex and the sensory cortex and less reactivity in the motor cortex. This finding is not exclusive for this pathology, and other diseases express it as schizophrenia, autoimmune diseases, and neoplasms [7].

The HTLV-I virus is associated with systemic inflammatory diseases such as uveitis, arthritis, Sjogren's syndrome, and pulmonary alveolitis; in the nervous system, the involvement of the central and peripheral nervous system with clinical spectra is reported from mild cognitive impairment, leukoencephalopathy, amyotrophic lateral sclerosis, myelopathy, peripheral neuropathy, and polymyositis [4-6].

Treatment options follow the guidelines that apply to patients who have TSP/HAM syndrome, with supportive measures and symptomatic treatment including steroids to modulate the inflammatory response. The different workgroups have attracted controversies regarding their use and have reported improvement in response rates and relief of symptoms in patients with high viral loads [2]. The results of a recent study that evaluated patients for several years highlighted the improvement in disability scales in the group that took prednisone in both the short and long term when compared with untreated patients [8,9]. Initial schemes involve methylprednisolone 100 mg IV per day for three days, followed by oral prednisone 15 mg per day [2,8,9]. There are alternative proposals with anti-inflammatory drugs such as cyclosporine, pentoxifylline, danazol, and interferons [8,10]. Flow charts of treatment for the disease show a regular response to steroids or improvement in progression and propose alternatives to continue with interferon, cyclosporin A, danazol, and vitamin C [10]. New therapeutic options that focus on eliminating infected cells and reducing the proviral load have emerged; the antigens of the virus are not recognized by the immune system and drugs such as valproic acid, by the mechanism of action that inhibits the enzyme histone deacetylase, induce the expression of the proviral gene HTLV-I, generating recognition by the immune system and leading to cytotoxic-type response [8,10]. Regarding the goal of reducing viral load, some of the work done have been controversial. There are reports of good results with the combination of valproic acid and zidovudine-type antivirals that focus on the prevention of neo-infection. It is proposed for preventive purposes in asymptomatic carrier patients and to limit the increase in proviral load, which is the best-defined risk factor to develop the syndrome [8,10]. The drug mogamulizumab (antibody against chemokine receptor CCR4, which is used in the treatment of adult T cell leukemia-lymphoma associated with HTLV-I retrovirus) has been found to reduce viral load, the proliferation of T and B cells expressing CCR4, and the production of inflammatory cytokines. Recent work has focused on assessing the usefulness of this neurological syndrome [8-11].

## Conclusions

The HTLV-I virus is associated with central and peripheral nervous system involvement, a relationship that has been established by the increased expression of HERV-K in the brain tissue of patients with ALS. The treatment of the condition focuses on symptomatic management and eliminating infected cells to reduce the viral load by using drugs such as steroids, valproic acid, the antiviral zidovudine, and mogamulizumab (monoclonal antibody to the T cell CC chemokine receptor 4).
